# Correlative humoral and cellular immunity to genetically attenuated malaria parasites in humans

**DOI:** 10.1016/j.isci.2025.112589

**Published:** 2025-05-05

**Authors:** Emil Colstrup, Rie Nakajima, Jelte M.M. Krol, Olivia A.C. Lamers, Rafael R. de Assis, Aarti Jain, Algis Jasinskas, Eva Iliopoulou, Helena M. de Bes-Roeleveld, Blandine M.D. Franke-Fayard, Meta Roestenberg, Philip L. Felgner, Rajagopal Murugan

**Affiliations:** 1 Leiden University Center for Infectious Diseases, Leiden University Medical Center, Leiden, the Netherlands; 2 Department of Physiology and Biophysics, School of Medicine, University of California, Irvine, Irvine, CA, USA

**Keywords:** Immunity, Microbiology parasite

## Abstract

Malaria caused by *Plasmodium falciparum* remains one of the major infectious diseases with a high burden in Sub-Saharan Africa. In spite of the advancements made in vaccine development and implementation in endemic countries, sterile and durable protection has not been achieved. Recently, we have shown the superior protective capacity of whole sporozoites attenuated to arrest late (GA2) but not early (GA1) during the liver stage development in a controlled human malaria infection study. Here we report the breadth of antigens targeted by hitherto understudied parasite liver stage immunity and convey the coherence between humoral and cellular immunity observed in our clinical study. Our findings uncover the underlying immunogenic differences between early- and late-liver stage arresting parasites and identify key liver stage antigens for future vaccine development focused on inducing sterile immunity to malaria.

## Highlights

• Late-liver stage arresting human malaria parasite GA2 induces potent immunity
• Antigenic breadth and intensity of humoral immunity increases with parasite exposure
• GA2 induces pro-inflammatory polyfunctional effector memory CD4 T cells
• Effector memory T cell response synergizes with antigenic breath of humoral immunity

## Graphical abstract

**Figure:**
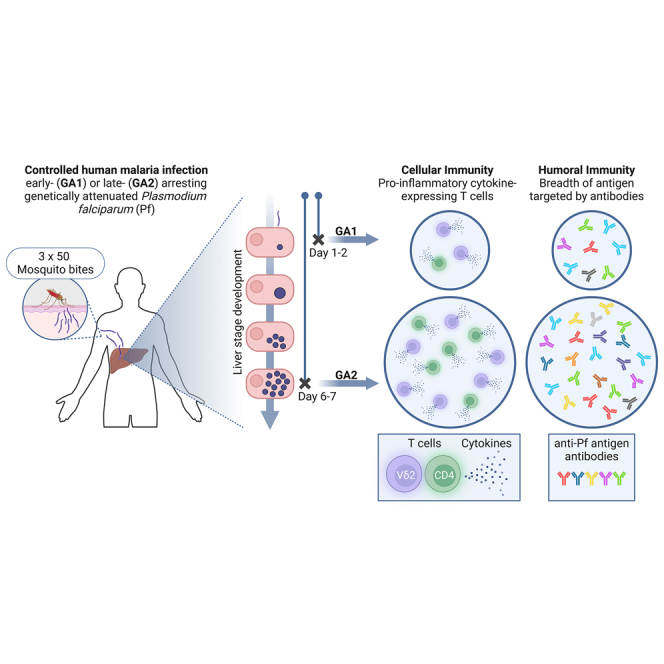

## Introduction

Malaria is one of the major life-threatening infectious diseases worldwide, causing 608,000 deaths and 249 million infections in 2023.^1^ Among the five human infective species, *Plasmodium falciparum* (Pf) remains the cause of highest disease morbidity and mortality, particularly in low- and middle-income countries in Sub-Saharan Africa. Despite major efforts on disease control and vaccine development, reduction in malaria cases has stalled in recent years, challenging World Health Organization (WHO) global health and disease eradication programs. Currently, there are two WHO-approved malaria vaccines available for clinical use: RTS,S/AS01 (Mosquirix) and R21/Matrix-M.^2^^,^^3^^,^^4^ While the vaccines significantly reduce the incidence of clinical malaria, the protective immunity tends to wane over time.^5^^,^^6^

Within 1–2 h post-infected mosquito bite, Pf sporozoites reach the liver and establish intracellular infection within host hepatocytes, where they develop during the next 7 days into symptomatic erythrocyte infective merozoites, and later on into transmission driving gametocytes. Developing effective malaria vaccines remains a considerable challenge due to the complex life cycle of Pf, its substantial genetic diversity, and sophisticated immune evasion mechanisms.^7^^,^^8^ Both cellular and humoral immune responses are associated with protection against malaria, but the precise antigens that, either by themselves or collectively can confer sterile and durable protection are not yet fully known.^9^^,^^10^ Moreover, the parasite’s large genome, encoding over 5,300 antigens, further complicates identification of key antigens targeted by protective immunity,^11^^,^^12^ with only a fraction evaluated as vaccine candidates.^13^ In contrast to antigens expressed during early sporozoite, late merozoite, and gametocyte stages, which were advanced into vaccine designs, liver stage antigens remain largely uncharacterized, leaving a major blockade in pan-stage vaccine designing approach.^4^^,^^14^^,^^15^^,^^16^

Immunization with whole sporozoites offers a strong possibility to assess overall protective capacity and discovery of immunogenic antigens. Such approaches utilize distinct attenuation strategies that arrest parasite development in the human host at specific stages. While radiation-attenuated sporozoites (RAS) arrest soon after infecting hepatocytes,^17^ chemo-attenuation by chloroquine chemoprophylaxis (CPS) allows for full parasite maturation in the liver and subsequent clearance in the blood.^18^^,^^19^ Recently, we have shown that targeted genetic attenuation arrests the malaria parasites at specific development stages in the liver.^20^^,^^21^ These genetically attenuated parasites (GAPs) offer greater antigenic exposure compared to RAS while arresting precisely before the onset of symptomatic blood stage parasites. We demonstrated that malaria parasites genetically attenuated to arrest late, but not early, during the liver stage development show high protective capacity in malaria naive-humans.^21^ In this clinical trial, sterile protection was achieved in 8 of 9 participants immunized with a late-arresting GA2 (*PfΔmei2*) parasite, compared to 1 of 8 participants immunized with an early-arresting GA1 (*PfΔb9Δslarp*) parasite.^21^

Progress in high-density whole-proteome microarrays allows for rapid analysis of humoral responses to infectious diseases, supporting both vaccine antigen identification and serodiagnostic antigen discovery.^22^^,^^23^^,^^24^ In this study, we made use of a Pf proteome microarray coated with the selected set of Pf antigens reported to associate with protection in previous studies.^25^ Using the unique clinical trial samples, here we report the distinct immunogenicity of GA1 and GA2 parasites by analyzing the humoral antibody and cellular responses. Our work uncovered antigens targeted by the immune response to late-liver stage arresting parasites and shows that the overall breadth of the humoral immunity correlates with Pf-specific cellular immunity in the clinical trial participants.

## Results

**Figure 1:**
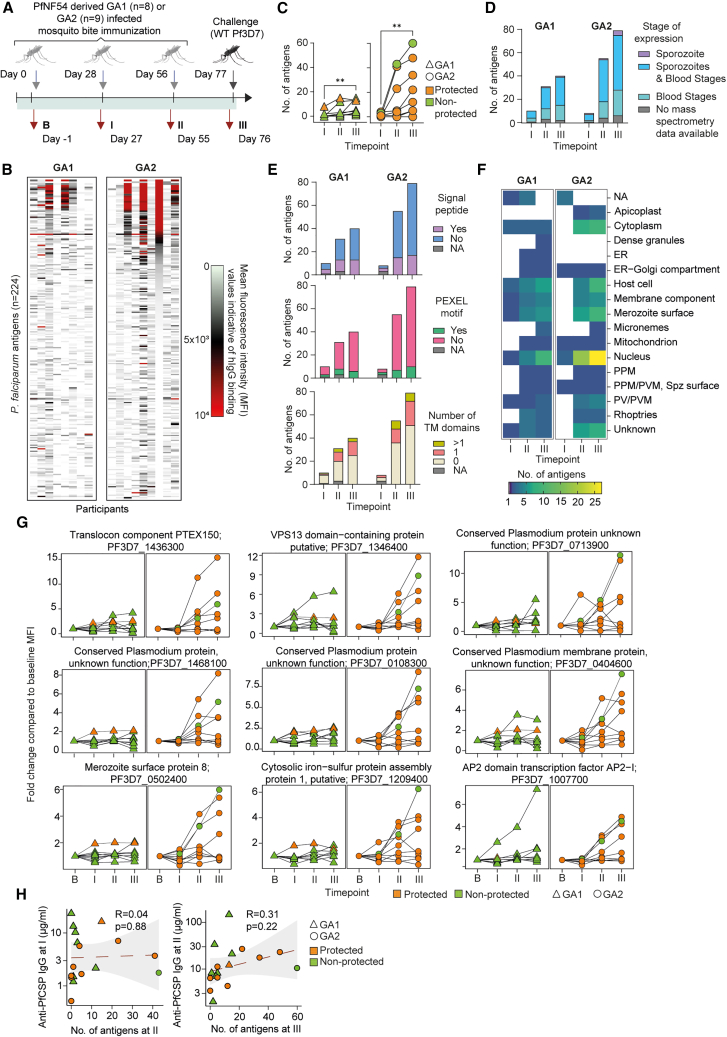
Immunization with the late liver stage arresting parasite induces a stronger and broader humoral response compared to early-arresting parasite (A) A schematic overview of the clinical trial where participants were immunized three times with mosquito bites (MBs) infected with either early (GA1) or late (GA2) arresting Pf parasites, followed by a challenge with wild type (WT) Pf.^21^ (B) IgG binding values in plasma collected at (III) from microarray coated with 262 peptides covering 224 unique antigens. (C) The number of targeted antigens in GA1-MBs and GA2-MBs participants across three time points. Statistical analysis was performed by the Friedman test and the *p* values were indicated. (D) Predicted expression stage of antigens targeted upon GA1-MBs and GA2-MBs immunization. (E) Predicted presence of signal peptide (top), Pf-specific PEXEL motifs (middle), and the number of transmembrane domains (bottom) among the antigens targeted upon GA1-MBs and GA2-MBs immunization. (F) Predicted location of targeted antigens after GA1-MBs and GA2-MBs. (G) The MFI fold change compared to baseline (B) of Pf antigens with higher seroprevalence in GA2-MBs compared to GA1-MBs following three immunizations. (H) Pearson correlations between targeted antigen counts at the indicated immunization time point and α-PfCSP antibody titers following the previous immunization. See also Figure S2 and Tables S1, S2–S5, S6, and S7.

To investigate the humoral immunity induced against the GAPs that arrest early (GA1) or late (GA2) during the liver stage development, we collected plasma samples at baseline (B), 27 days post first (I) and second (II) immunizations and 20 days post third (III) immunization (Figure 1A). We used protein microarrays consisting of 262 Pf antigen peptides, representing 224 unique antigens covering both pre-erythrocytic and blood stages based on published studies of controlled^24^^,^^26^ and natural infection.^22^^,^^27^^,^^28^^,^^29^ We measured plasma IgG antibodies specific to each antigen in the clinical trial participants across four time points. Heatmaps depicting the bound IgG levels as mean fluorescence intensity (MFI) values indicate the distinct humoral immunity induced by GA1-mosquito bites (MBs) and GA2-MBs in clinical trial participants (Figure 1B; Table S1).

To qualitatively assess the response and account for inter-individual background noise, we pooled the MFI values from the time points B and III for each participant separately and defined the threshold for positive signal using a bimodal distribution calculated by mixtools,^30^ a computational R function (Figure S1A). By identifying and enumerating Pf antigens with IgG MFI values higher than the threshold, we observed an increase in the total number of antigens targeted over three Pf exposures (Figure 1C; Table S2). Notably, humoral immunity to GA2-MBs targeted more antigens compared to GA1-MBs. As GA1 and GA2 parasites differ in how far they progress into the liver stage development, we analyzed the expression stages of the antigens targeted by humoral immunity. Using the tandem mass spectrometry information available for the antigens from PlasmoDB,^11^ we found that a large proportion of antigens were expressed during the sporozoite and blood stages (Figure 1D; Table S3). However, GA2-MBs immunization targeted a higher number of antigens expressed either exclusively in the blood stage or also during the sporozoite stage over three Pf exposures compared to GA1-MBs.

To understand whether the antibody response preferentially targeted antigens that are secreted to the parasite periphery, we investigated the presence of signal peptides using PlasmoDB and SignalP6.0 (Figure 1E; Table S4).^11^^,^^31^ In addition, we performed screening for *Plasmodium* export element (PEXEL) motifs that may signify potential for protein translocation to or beyond the parasitophorous vacuole membrane (PVM) and thus may indicate localization at the parasite periphery or host cell cytoplasm. Furthermore, we assessed the presence of transmembrane domains in the antigens to predict their localization on the parasite surface, PVM, secretory network vesicles or host membranes. Interestingly, most antigens targeted by the response against GA1-MBs and GA2-MBs did not possess the signatures for secretion and membrane localization, suggesting an intracellular localization profile. Notably, utilizing PlasmoDB and Gene Ontology terminology, we found that the majority of antigens were predicted to have nuclear or cytoplasmic localization, whereas a subset of membrane-bound antigens were targeted (Figure 1F; Table S5).

To identify response characteristics influenced by the parasite attenuation stage, we estimated the seroprevalence of antigens during GA1-MBs and GA2-MBs immunizations and compared to the previously reported seroprevalence in naive individuals upon exposure to three unattenuated wild-type (WT) PfNF54 infectious MBs under CPS^21^^,^^26^ (Figure S1B). The targeted antigens and their seroprevalence in GA2-MBs resembled that of CPS more than GA1-MBs, suggesting an overlap in antigens expressed and exposed to the immune system in GA2 and WT parasites during the late liver stage. In spite of the liver stage arresting phenotype, we observed several blood stage antigens being targeted by the GA2-MBs response, but not by the GA1-MBs response, such as merozoite surface protein 8 (PF3D7_0502400). This indicates that these antigens are expressed in both the late-liver stage and the blood stage. We identified key Pf antigens that were preferentially targeted during GA2-MBs in comparison to GA1-MBs such as translocon component PTEX150 (PF3D7_1436300), the VPS13 domain-containing protein, putative (PF3D7_1346400), and the conserved *Plasmodium* protein (PF3D7_0713900; Figures 1G and S2; Table S6). Although the vast majority of the targeted antigens have a predicted nuclear or cytoplasmic localization, some of the key antigens with high seroprevalence in GA2-MBs showed membrane localization, indicating the distinct feature of response against late liver stage arresting GA2 parasites. Moreover, we observed a clear booster effect in the IgG antibody levels against these antigens upon three GA2-MBs but not GA1-MBs, suggesting the formation and reactivation of memory B cells against the antigens. Overall, while increasing the total levels of antibodies targeted toward the antigens over three immunizations, high responders in GA2-MBs cohort continued to target additional number of antigens after each immunization, indicating both amplification and diversification of the response (Figures 1C and 1G). It is also noteworthy that 27 out of the 85 targeted antigens are *Plasmodium* proteins with unknown functions, 23 of which are annotated as “conserved,” highlighting the critical need to better understand their roles in liver stage development. Additionally, 29 of the 85 targeted antigens are classified as putative, suggesting their function and role during liver stage development are not accurately annotated. To address the heterogeneity of the GA2-MBs response, we asked whether having strong anti-sporozoite antibody levels would limit the response against liver stage antigens. However, we observed no correlation between the pre-MBs anti-Pf circumsporozoite protein (PfCSP) antibody levels and the number of antigens targeted post-MBs during both second and third immunization, suggesting the observed GA2-MBs response heterogeneity is independent of anti-sporozoite immunity (Figure 1H; Table S7). Overall, our results show that GA2-MBs induced stronger and broader levels of antibodies targeting several late-liver and blood stage antigens compared to GA1-MBs. While the majority of the targeted antigens are predicted to be expressed intracellularly, a subset of membrane-bound antigens were also targeted by GA2-MBs response.

**Figure 2:**
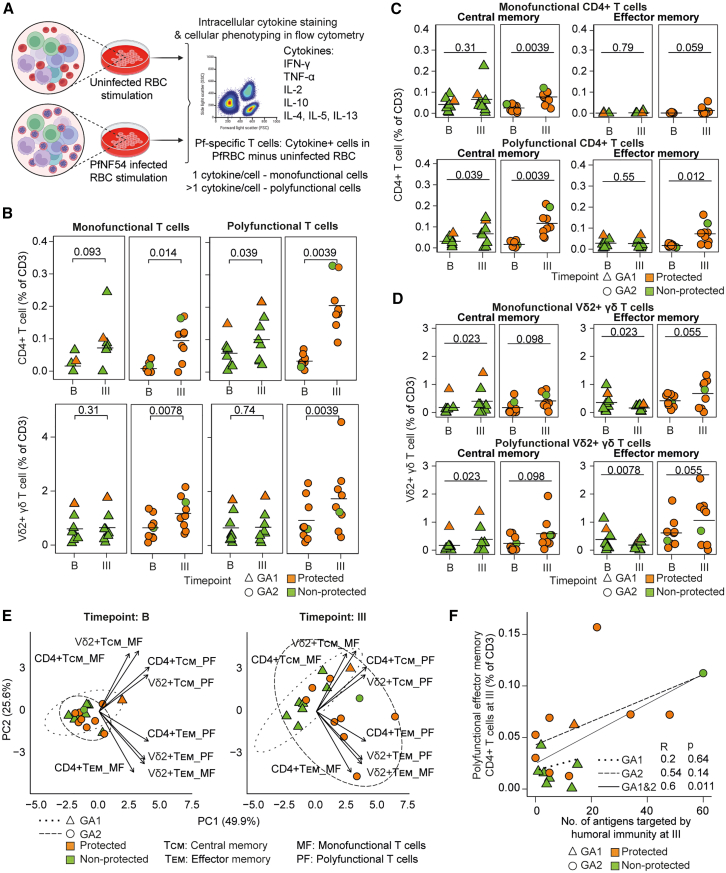
Pf-specific polyfunctional effector memory T cell response correlates with the humoral response (A) Experimental design of stimulating PBMCs with Pf-infected or uninfected erythrocytes to detect Pf-specific cellular responses in immunization groups. (B) Frequency of monofunctional (1 cytokine/cell) and polyfunctional (>1 cytokine/cell) CD4^+^ and Vδ2^+^ γδ T cells after three immunizations (III) compared to the baseline (B). (C and D) Frequencies of cellular responses defined as central (CCR7^+^CD45RA^−^, T_CM_) and effector (CCR7^−^CD45RA^−^, T_EM_) memory T cells as previously reported among CD4^+^ (C) and Vδ2^+^ γδ (D) T cells.^21^ (E) Principal component analysis of mono- and polyfunctional CD4^+^ and Vδ2^+^ γδ T_CM_ and T_EM_ frequencies at both baseline and following three immunizations. (F) Pearson correlation of polyfunctional CD4^+^ T_EM_ with the number of targeted antigens following three immunizations. (B–D) Statistical analysis was performed by non-parametric paired t test with *p* values indicated in the panels. Black horizontal lines indicate arithmetic mean. See also Figures S4A and S4B, and Tables S8, S9, and S10.

We previously reported the stronger cellular immunity of GA2-MBs compared to GA1-MBs by measuring the Pf-specific CD4^+^ and Vδ2^+^ γδ but not CD8^+^ T cells upon stimulating the peripheral blood mononuclear cells (PBMCs) with Pf-infected and uninfected erythrocytes (Figure 2A).^21^ By comparing the cellular immunity at baseline and after three exposures, we observed that both GA1-and GA2-MBs induced monofunctional and polyfunctional T cells that express single or more than one cytokines, respectively (Figure 2B; Table S8). Notably, our stimulation approach induced relatively higher frequencies of type-1 (interferon [IFN]-γ, tumor necrosis factor alpha [TNF-α], and interleukin [IL]-2) compared to type-2 (IL-10, IL-4, IL-5, and IL-15) cytokine expressing CD4^+^ and Vδ2^+^ γδ T cells among both mono- and polyfunctional T cells (Figure S3). We observed preferential expression of IFN-γ and TNF-α in Vδ2^+^ γδ T cells, whereas CD4^+^ T cells expressed all three pro-inflammatory cytokines as either mono- or polyfunctional T cells (Figure S3). While polyfunctional CD4^+^ T cells are significantly induced by both GA1- and GA2-MBs, only GA2-MBs induced polyfunctional Vδ2^+^ γδ T cells.

To identify the distinguishing features of cellular responses, we defined the phenotypic status of Pf-specific T cells as central memory (T_CM_: CCR7^+^CD45RA^−^) and effectory memory (T_EM_: CCR7^−^CD45RA^−^) as previously reported (Figures 2C and 2D; Table S9).^21^ Principal component analysis based on the frequencies of Pf-specific T cells indicated that the effector memory compartment in both CD4^+^ and Vδ2^+^ γδ T cells best distinguished the GA2-MBs induced cellular immunity from that of GA1-MBs, independent of mono- and polyfunctional status of the responding cells (Figure 2E; Table S9). To integrate our findings from the cellular immunity with humoral response measured at the level of individual participants, we compared the frequencies of Pf-specific T cells with the total number of antigens targeted at time point III (Figures 2F, S4A, and S4B; Table S10). Our analysis revealed a stronger positive correlation between polyfunctional effector memory CD4^+^ T cells and the number of antigens targeted in GA2-MBs compared to GA1-MBs, suggesting the overall coherence between cellular and humoral responses during immunization with whole sporozoites genetically attenuated to arrest at late-liver stage development.

## Discussion

Once the Pf sporozoite invades a human hepatocyte, the parasite undergoes full maturation inside the infected hepatocyte until the hepatocyte ruptures and releases as mature merozoites into the bloodstream. Hence, protection during the liver stage development most likely is mediated by cellular but not humoral immunity. However, due to the lack of knowledge of precise antigens expressed during liver stage development in Pf, key antigens that drive the immunogenicity and protective capacity during the parasite liver stage remain unknown.^32^^,^^33^^,^^34^ By studying the humoral response, here we report a list of antigens that are preferentially targeted by the B cells following GA1-MBs and GA2-MBs immunization. Given the stronger overlap of antigens targeted by GA2-MBs with CPS than GA1-MBs, the antigens we identified most likely are expressed during the late- but not early-liver stage of the parasite development. These antigens are likely exposed to the immune system either directly during hepatocyte rupture or through acquisition by antigen-presenting cells such as Kupffer cells.^35^^,^^36^ Furthermore, comparable protective levels achieved by GA2-MBs and CPS clinical studies indicate that the response to antigens exposed during the late-liver stage development sufficiently captures the protective response induced in CPS studies.^18^^,^^19^

The parasite liver stage development remains a black box as to the antigens that are specifically expressed during this stage, host-parasite interactive elements that are essential for parasite survival and development, and importantly, the antigens that are likely exposed to and targeted by the immune system to establish sterile protection. Studies in murine models are limited by the difference in development duration and antigen repertoire present between murine parasites such as *P. berghei* (Pb) and *P. yoelii* and human parasite *P. falciparum*.^37^^,^^38^^,^^39^ Nevertheless, these studies indicate the prominent role of monocyte-derived CD11c^+^ cells in acquiring infected hepatocytes and priming liver stage antigen-specific CD8^+^ T cells.^40^ However, the parallel role of such innate activation to trigger B cell response is not reported. Given the stronger and broader antibody response against Pf antigens in GA2-MBs in comparison to GA1-MBs, we believe that the antigen-presenting cells such as previously reported CD11c^+^ cells trigger B cell responses in the hepatic draining lymph node following the acquisition of infected hepatocytes. This would also explain the stronger polyfunctional CD4^+^ T cell response in GA2-MBs compared to GA1-MBs when stimulated with Pf-infected red blood cell (PfRBC) lysates, which can be used as a surrogate for late liver stage antigens. However, whether the antigens targeted by the humoral response elicited polyfunctional CD4^+^ T cells remains to be studied. Future studies should address the potential of the antigens reported here to elicit liver-resident CD4^+^ and CD8^+^ cellular immunity and their capacity to establish protection against malaria. Baseline samples stimulated with PfRBC lysates indicate potential differences in effector memory T cells. Influence of baseline cellular immune status in shaping the response to GA2-immunization needs to be addressed in-depth in future studies.

As superior protective levels of GA2-MBs compared to GA1-MBs are based on the immunogenicity of the parasite resulting from late-liver stage development, the immunogenic antigens that we report here provide a starting point to study the potential of liver stage antigens in eliciting cellular immunity and assess their protective efficacy. Current malaria vaccine development approaches are based on inducing high levels of protective immunity against sporozoites (PfCSP),^2^^,^^4^ blood stage (Rh5 and MSP1)^16^^,^^41^ and gametocyte stage (Pfs48/45, Pfs25, and Pfs230).^42^ Studies combining Pb sporozoites with liver stage antigens show superior protective capacity in murine models upon challenge with Pb.^43^^,^^44^ However, whether the ortholog Pf versions of the Pb liver stage antigens induce similar protective capacity remains unknown. Our study identifies a collection of antigens that are exclusively targeted by the humoral response of late-liver stage arresting parasite, hence opening new directions for liver stage targeted subunit malaria vaccine developments.

### Limitations of the study

Our results are limited to plasma antibody binding to Pf proteome representing protein sub-domains generated by *in vitro* transcription and translation platform. Antibody binding identified to the key target antigens needs to be validated in future studies using full-length protein expression platforms or in the native context in liver and blood-stage parasites. Given that only a single GA1-MBs and GA2-MBs immunized participant remained protected and non-protected, respectively, association between the reported immune response and clinical protection remains unclear. Future studies with a large cohort size needs to be performed to assess the immune correlates of protection. Sample size per cohort in the clinical study limits assessing the influence of demographic factors such as age and gender on the observed immunological responses. As our cellular analyses relied on stimulation of PBMCs by Pf infected erythrocytes, potential presence of liver-resident Pf-specific CD8^+^ T cells remains uncharacterized in this work.

## Acknowledgments

The authors would like to acknowledge the clinical trial participants for their contribution to this study. The trial received support from the 10.13039/501100005040Bontius Foundation. This research has received funding from 10.13039/501100001826ZonMw under VIDI grant no. 09150172010035, the 10.13039/501100000780European Union under 10.13039/100010663ERC St grant agreement no. 101075876, and 10.13039/501100007442Gratama Stichting/Leids Universiteits Fonds no. 2023-09/W233054-2. R.M. was supported by the 10.13039/501100007601European Union’s Horizon 2020 research and innovation program under the Marie Skłodowska-Curie grant (101109084).

## Author contributions

All authors read and approved the final manuscript. E.C. performed experiments and analyzed the data. R.N., R.R.d.A., A. Jain, and A. Jasinskas performed the microarray experiments. E.C., O.A.C.L., E.I., and H.M.d.B.-R. performed cellular and humoral immune assessment experiments. J.M.M.K. provided characterization of antigens identified in this study. B.M.D.F.-F., M.R., P.L.F., and R.M designed the study. M.R. and R.M. acquired funding. R.M. performed data analyses and interpretation. Original draft of the manuscript was written by E.C. and R.M. with inputs from all authors.

## Declaration of interests

The authors declare no competing interests.

## STAR★Methods

### Key resources table

**Table:** 

REAGENT or RESOURCE	SOURCE	IDENTIFIER
**Antibodies**		

Alexa Fluor® conjugated goat anti-human IgG Fc fragment-specific secondary antibody	Jackson ImmunoResearch	AB_2632452

**Chemicals, peptides, and recombinant proteins**		

Polypeptides from *in vitro* transcription and translation (IVTT) reactions.	Phil Felgner, University of California, Irvine, CA	N/A

**Deposited data**		

Cellular data upon PfRBC and unRBC stimulation of PBMCs	Lamers et al.^21^ NEJM	N/A
PlasmoDB for Pf proteome localization prediction	Aurrecoechea et al.^11^	N/A

**Recombinant DNA**		

T7 promoter plasmids encoding protein for IVTT reaction	Felgner et al.^24^ PNAS	N/A

**Software and algorithms**		

ProScanArray Express	PerkinElmer LAS, Waltham, MA	N/A
FlowJo	FlowJo, LLC	N/A
GraphPad	Prism	10.2.3
R and RStudio	R statistical program	4.3.3
SignalP-6.0	SignalP 6.0 - DTU Health Tech - Bioinformatic Services	6.0

### Experimental model and study participant details

#### Ethics

The clinical trial protocol was approved by the Dutch Central Committee for Research Involving Human Subjects (CCMO file number: NL75577.000.21) and registered at ClinicalTrials.gov (NCT04577066). Licence from the Dutch Gene Therapy Office was obtained to perform the trial and a data and safety monitoring committee oversaw the trial. Written informed consent was obtained from all trial participants.

#### Clinical study

In the current study, 20 healthy malaria-naïve Dutch participants received bites from 50 mosquitoes, of which 8 participants received bites infected with early arresting GA1 parasites, 9 participants received bites infected with late arresting GA2 parasites, and 3 participants received uninfected mosquitoe bites.^21^ Participants were immunized three times at 4-week intervals. Among the participants, 1 out of 8 who received GA1 showed protection, while 8 out of 9 receiving GA2 were protected, and all 3 placebos were unprotected.^21^ Blood and plasma samples were collected 1 day prior to each immunization (I, II, III) and prior to challenge through controlled human malaria infection (CHMI) with WT Pf 3D7 (Figure 1A). Protection is characterized by the absence of detectable parasitemia as measured in Pf qPCR throughout the 21-day daily follow-up period after challenge with WT Pf 3D7.

### Additional resources

This study involves a clinical trial registered at ClinicalTrials.gov with the registration number NCT04577066.

### Method details

#### Construction and probing of the microarray

A selected Pf3D7 proteome microarray was constructed at University of California, Irvine, CA. Sequence-verified polypeptides were printed as IVTT reactions, following previously established methods.^45^ The T7 promoter plasmids used for encoding each protein in the IVTT reactions were acquired through a high-throughput cloning technique.^46^ Quality control of the array slides was carried out as described in earlier studies,^24^ yielding over 97% protein expression efficiency from the spotted IVTT reactions.

Microarray slides were first blocked with the protein array blocking buffer (GVS S.p.A., Zola Predosa, Italy) for 30 minutes. Plasma samples were diluted 1:100 in protein array blocking buffer with an added 10% (vol/vol) *E. coli* BL21(DE3) lysate (Genscript, Piscataway, NJ) and were incubated at room temperature for 30 minutes. The lysate was used to block anti-*E. coli* antibodies in the plasma samples, hence minimizing non-specific background binding. Thereafter, microarray slides were incubated with diluted plasma samples overnight at 4°C.

Antibody binding was detected using an Alexa Fluor conjugated goat anti-human IgG Fc fragment-specific secondary antibody (Jackson ImmunoResearch, West Grove, PA), diluted 1:200 in blocking buffer. The microarrays were scanned using a TinyArray imager,^47^ and spot intensities were analyzed using the ProScanArray Express system (PerkinElmer LAS, Waltham, MA), with spot-specific background correction applied.

Raw data from the scans were expressed as the mean pixel intensity within the printed spots, with surrounding background intensities subtracted for each protein or control. Negative control spots, known as IVTT or “no-DNA” controls, consisted of IVTT reaction mixtures without plasmid templates. The average intensity of these control spots was subtracted from the experimental spots. Correlation analysis of arrays processed on different days and from distinct slide batches confirmed the reproducibility of the slide printing and probing.

#### Data normalization and analysis

Data normalization is performed in two steps. First, using the whole dataset, the control spots are normalized using the Quantile Normalization method (“preprocessCore” package (Version 1.50.0)). For each sample, a rescaling factor is calculated by dividing the sum of the normalized control spots by the sum of the raw (non-normalized) control spots of the corresponding sample. The resulting factor is then multiplied by the reactivity of each spot resulting in a rescaled data frame. To address variations in baseline antibody levels among immunized individuals and to measure specific antibodies induced by GAP immunization, a computational method implemented with the R package mixtools,^30^ was used to define binding thresholds for individual participants. The two-component mixture model was developed using a participant’s bound antibody levels at both the baseline (B) and after three immunization (III) time points across all 262 peptides. This model differentiates between positive and negative values based on the bound antibody distributions, by means of defining the cutoff point set as the mean of the negative antibody signal distribution plus three times the standard deviation as applied in the previous study.^26^ Additionally, seropositivity for Pf antigens was considered only when the bound antibody levels at a given time point post-immunization were at least 2-fold higher compared to that of baseline.

#### Pf-specific cellular immunity measurement

Cellular immunity was measured as described previously.^21^ In brief, PBMCs isolated from the clinical trial participants were stimulated with Pf-infected and uninfected erythrocytes for 24 hours. The cells were stained for specific cell surface markers for central (CCR7^+^CD45RA^−^, T_CM_) and effector (CCR7^+^CD45RA^−^, T_EM_) memory CD4^+^ and Vδ2^+^ γδ T cells. In addition, the cells were stained for intracellular cytokine markers for interferon-γ, tumor necrosis factor-α, interleukin (IL)-2, IL-4, IL-15 and IL-13.

### Quantification and statistical analysis

Data analyses were conducted in the R statistical program (version 4.3.3) and visualized through the ggplot2 R package.^48^^,^^49^ Additional plots were created in GraphPad Prism (10.2.3), using the built-in statistical tools for analysis. Statistical tests used were indicated in the corresponding figure legends and the actual *p* values were mentioned in the figure panels. Illustrations were created using BioRender.

## Supplemental information

Document S1. Figures S1–S4 and Tables S2, S3, S4, S5, and S7

Table S1. Microarray data, related to Figure 1B MFI values of microarray per antigen per participant at (III) minus (B) values

Table S6. Antigens with high seroprevalence upon GA2-MBs, related to Figure 1G Fold change MFI across time points (I, II, and III) compared to (B) MFI values

Table S8. Frequency of mono- and polyfunctional CD4^+^ and Vδ2^+^ γδ T cells, related to Figure 2B Frequency of monofunctional (1 cytokine/cell) and polyfunctional (>1 cytokine/cell) CD4^+^ and Vδ2^+^ γδ T cells after three immunizations (III) compared to the baseline (B)

Table S9. TCM and TEM frequencies among CD4^+^ and Vδ2^+^ γδ T cells, related to Figures 2C–2E Frequencies normalized to CD3^+^ population

Table S10. Correlations between targeted antigen counts and T cell population frequency, related to Figures 2F and S4
