# Supplementary material for: Correlative humoral and cellular immunity to genetically attenuated malaria parasites in humans
Source: iScience. 2025 May 5;28(6):112589. doi: 10.1016/j.isci.2025.112589 (PMC12145804; doi:10.1016/j.isci.2025.112589)
Supplement: Document S1. Figures S1–S4 and Tables S2, S3, S4, S5, and S7 [file mmc1.pdf]

iScience, Volume *28*

Supplemental information

Correlative humoral and cellular immunity to genetically attenuated malaria parasites in humans

Emil Colstrup, Rie Nakajima, Jelte M.M. Krol, Olivia A.C. Lamers, Rafael R. de Assis, Aarti Jain, Algis Jasinskas, Eva Iliopoulou, Helena M. de Bes-Roeleveld, Blandine M.D. Franke-Fayard, Meta Roestenberg, Philip L. Felgner, and Rajagopal Murugan

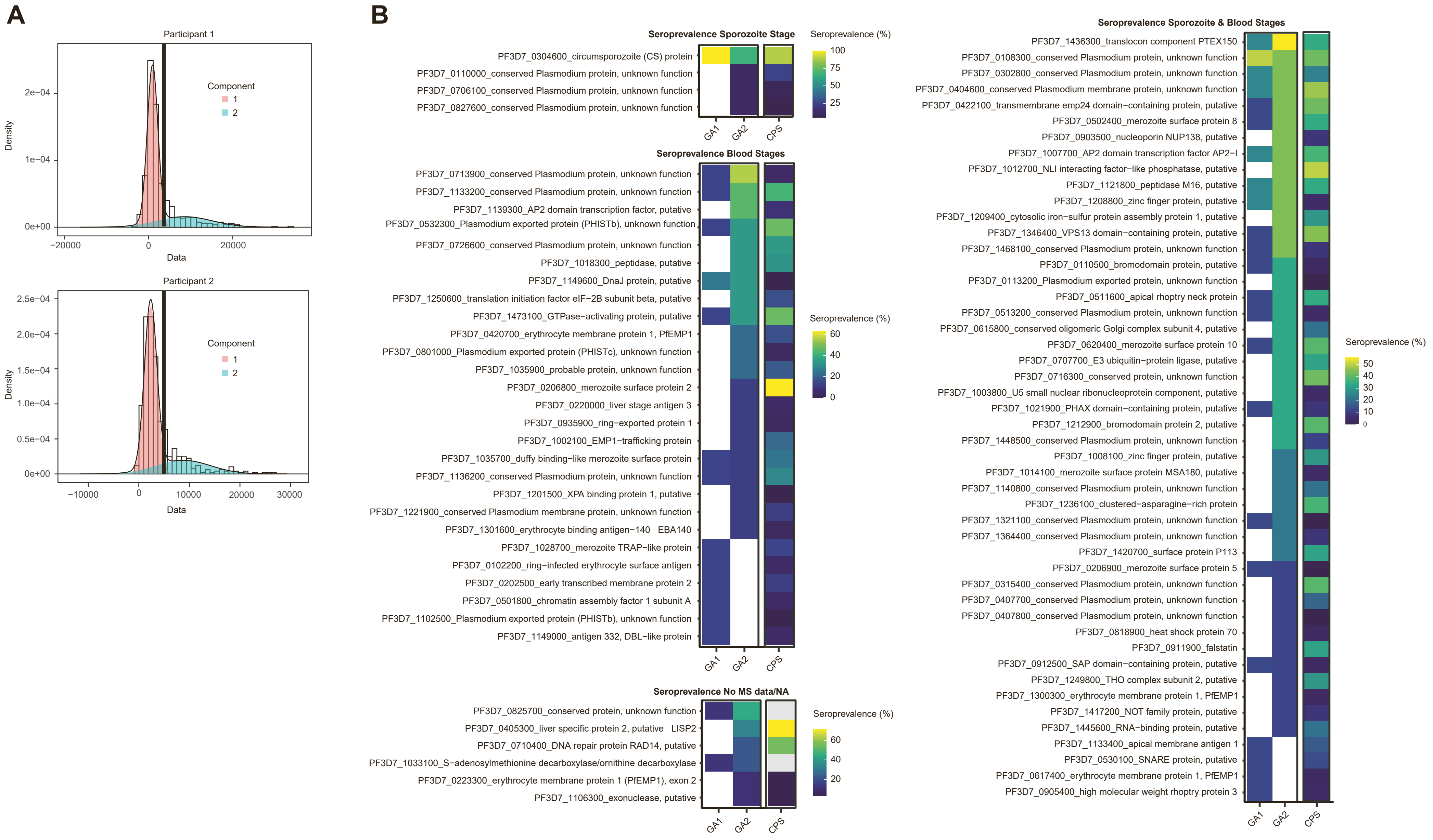

Figure S1: Microarray results representing seroprevalence of Pf-antigens targeted by different immunizations. A. Representative bimodal distribution plots of two clinical trial participants indicating negative and positive peaks and the dark black line indicates the threshold for antigen-positivity. **B.** Calculated seroprevalence of Pf antigens in GA1-MB and GA2-MB groups compared with CPS immunized participants published in Oberio et al. (27)

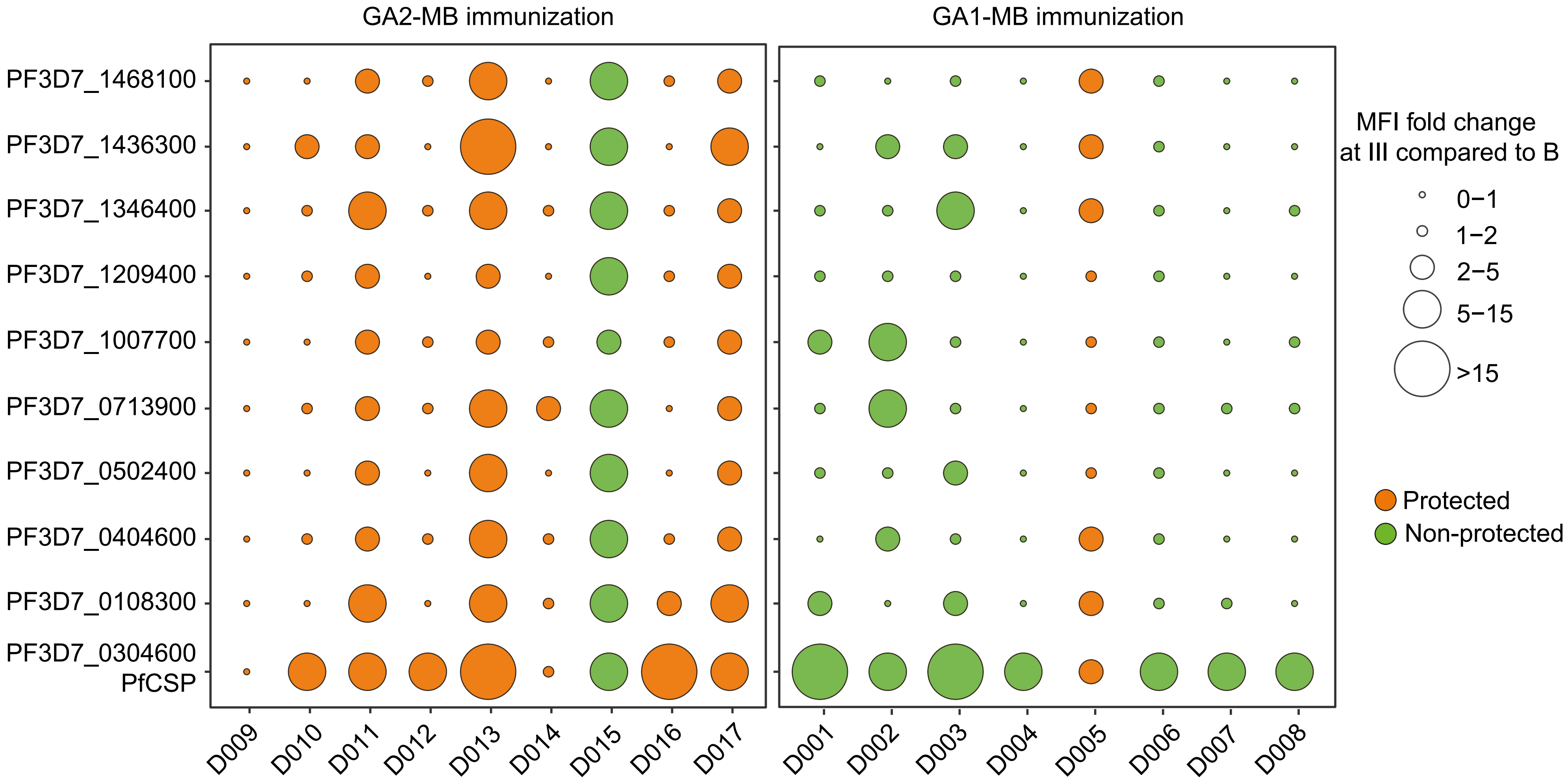

Figure S2: Increase in antibody levels targeting key Pf antigens after three immunizations (III) compared to baseline (B). Fold change in MFI values for the Pf antigens presented in Figure 1G compared the level of individual participants in GA1- and GA2-MB immunized cohorts (D001-D017). Antibody levels corresponding to PfCSP was shown for reference.

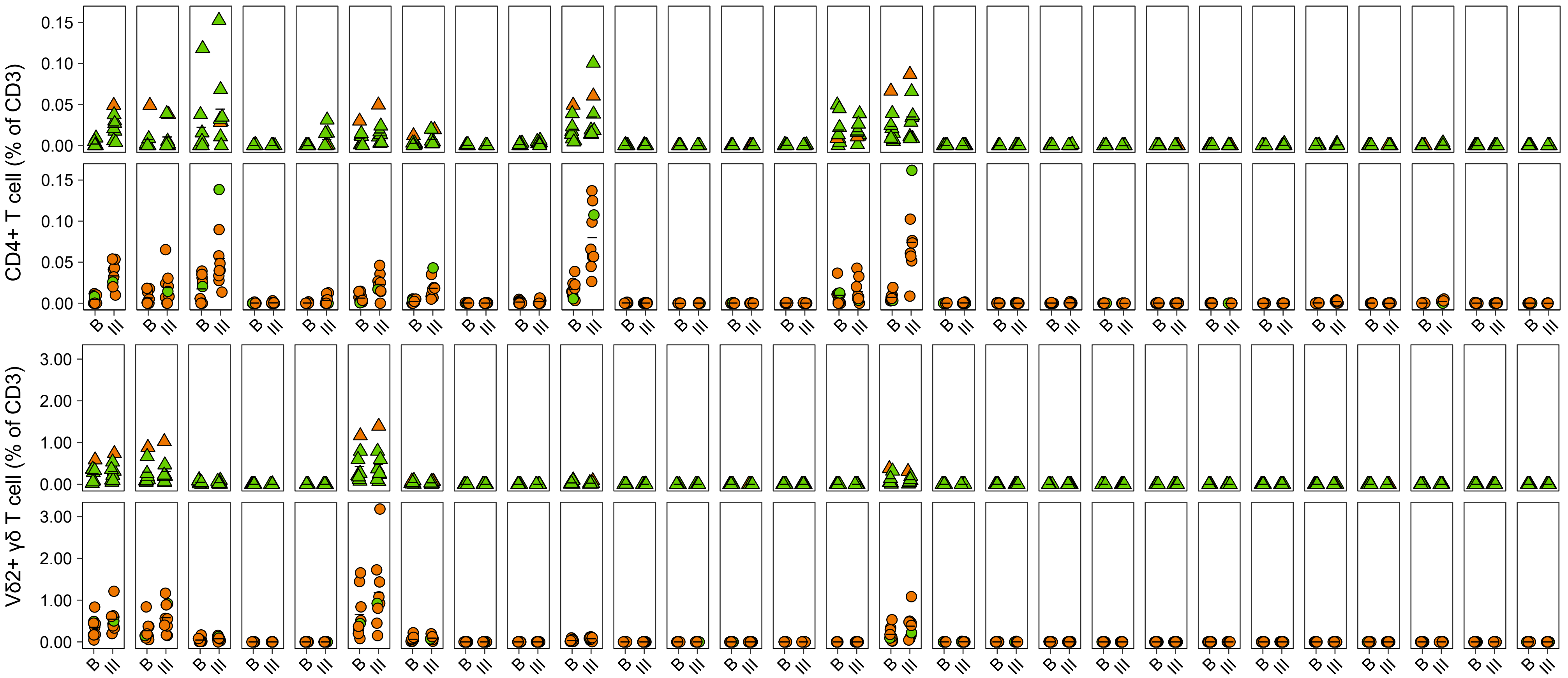

IFN-γ	+					+	+	+	+							+	+	+	+	+	+					+	+	
TNF-α		+				+				+	+	+				+	+	+				+	+	+		+	+	+
IL-2			+				+			+			+		+	+			+		+	+		+	+	+		+
IL-10				+				+			+		+	+				+		+	+	+	+		+		+	+
IL-4, 5, 13					+				+			+		+	+		+		+	+			+	+	+	+	+	+

△ GA1 ○ GA2 Protected Not protected

Figure S3: Cytokine expression in mono- and polyfunctional T cell response. Frequencies of CD4+ and Vδ2+ γδ T cells expressing the indicated cytokines (with the sign +). Black line indicates arithmetic mean.

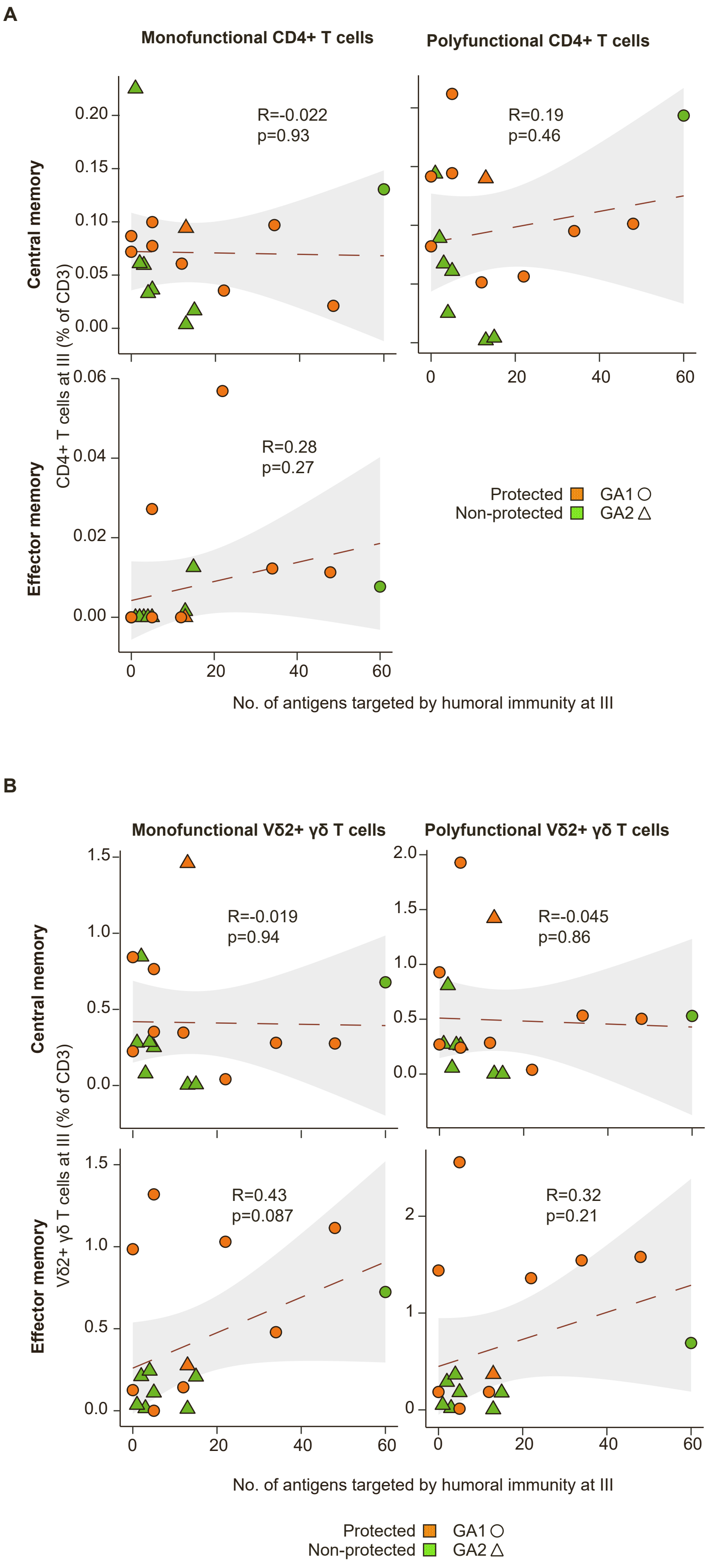

Figure S4: Correlation of Pf-specific cellular response to humoral immunity. A-B. Pearson correlation of mono- and polyfunctional CD4+ (A) and Vδ2+ γδ (B) TCM (top) and TEM (bottom) frequencies with the number of targeted antigens following three immunizations.

Table S1: Microarray data, related to Figure 1B. MFI values of microarray per antigen per participant at (III) minus (B) values.

See Table S1. Excel file containing additional data too large to fit in a PDF, related to Figure 1B

Table S2: Number of targeted antigens, related to Figure 1C. Number of antigens targeted across three timepoints among GA1-MB and GA2-MB groups.

GA1	Timepoint		
**Donor**	**I**	**II**	**III**
D001	0	0	3
D002	0	12	13
D003	0	1	15
D004	0	2	5
D005	8	15	13
D006	0	1	1
D007	0	1	2
D008	2	3	4

GA2	Timepoint		
**Donor**	**I**	**II**	**III**
D009	0	0	0
D010	1	1	5
D011	0	41	48
D012	4	5	12
D013	0	23	34
D014	0	0	0
D015	3	43	60
D016	0	0	5
D017	1	4	22

Table S3: Predicted expression stage of targeted antigens, related to Figure 1D.

Group	Timepoint	Sporozoite	Sporozoites & Blood Stages	Blood Stages	NA/No MS data
GA1	I	0	6	3	1
	II	1	18	9	3
	III	1	24	13	2
GA2	I	1	5	1	1
	II	1	36	14	4
	III	4	47	22	6

Table S4: Signal peptide, PEXEL motifs, and transmembrane domains, related to Figure 1E. Predicted presence of signal peptide, PEXEL motifs, and transmembrane domains among antigens targeted upon GA1-MB and GA2-MB immunization across three time points.

Signal peptide:

Group	Timepoint	Yes	No	N/A
GA1	I	4	5	1
	II	10	18	3
	III	13	27	0
GA2	I	3	2	3
	II	15	40	0
	III	17	62	0

PEXEL motif:

Group	Timepoint	Yes	No	N/A
GA1	I	2	7	1
	II	5	23	3
	III	6	34	0
GA2	I	2	3	3
	II	7	48	0
	III	10	69	0

Transmembrane domains:

Group	Timepoint	0	1	>1	N/A
GA1	I	7	1	1	1
	II	17	8	3	3
	III	25	12	3	0
GA2	I	3	2	0	3
	II	36	12	7	0
	III	51	21	7	0

Table S5: Expression localization, related to Figure 1F. Predicted location of targeted antigens after GA1-MB and GA2-MB.

	**GA1**			**GA2**		
**Loca on**	**I**	**II**	**III**	**I**	**II**	**III**
Apicoplast	0	0	0	0	1	2
Cytoplasm	2	2	2	0	7	9
Dense granules	0	0	1	0	0	0
ER	0	1	1	0	0	0
ER-Golgi intermediate compartment	0	1	1	1	1	1
Host cell	2	4	6	0	4	9
Membrane component	1	3	4	0	4	5
Merozoite surface	1	4	5	0	6	9
Micronemes	0	0	2	0	0	1
Mitochondrion	0	1	1	1	1	1
Nucleus	1	4	9	2	18	27
PPM	0	1	1	0	1	1
PPM/PVM, Sporozoite surface	0	1	1	1	1	1
PV/PVM	1	1	2	0	3	4
Rhoptries	0	3	2	0	2	2
Unknown	1	3	2	0	6	7
NA	1	3	0	3	0	0

Table S6: Antigens with high seroprevalence upon GA2-MB, related to Figure 1G. Fold change MFI across timepoints (I, II, III) compared to (B) MFI values.

See Table S6. Excel file containing additional data too large to fit in a PDF, related to Figure 1G

Table S7: Correlations between targted antigen counts and anti-PfCSP IgG antibodies, related to Figure 1H.

donor_id	Immunization	Protection	Number of Antigens (II)	anti-CSP IgG (I) (µg/ml)
D001	GA1	no	0	23,3
D002	GA1	no	12	2,2
D003	GA1	no	1	13,4
D004	GA1	no	2	10,3
D005	GA1	yes	15	16,6
D006	GA1	no	1	1,5
D007	GA1	no	1	1,2
D008	GA1	no	3	6,5
D009	GA2	yes	0	1,4
D010	GA2	yes	1	2,3
D011	GA2	yes	41	3,7
D012	GA2	yes	5	1,7
D013	GA2	yes	23	7,1
D014	GA2	yes	0	1,6
D015	GA2	no	43	1,8
D016	GA2	yes	0	0,5
D017	GA2	yes	4	5,7

donor_id	Immunization	Protection	Number of Antigens (III)	anti-CSP IgG (II) (µg/ml)
D001	GA1	no	3	33,8
D002	GA1	no	13	146,2
D003	GA1	no	15	21,2
D004	GA1	no	5	8,2
D005	GA1	yes	13	12,1
D006	GA1	no	1	8,2
D007	GA1	no	2	2,0
D008	GA1	no	4	7,0
D009	GA2	yes	0	6,4
D010	GA2	yes	5	11,3
D011	GA2	yes	48	23,1
D012	GA2	yes	12	4,3
D013	GA2	yes	34	17,6
D014	GA2	yes	0	3,4
D015	GA2	no	60	10,8
D016	GA2	yes	5	6,6
D017	GA2	yes	22	27,0

Table S8: Frequency of mono- and polyfunctional CD4$^+$ and Vδ2$^+$ γδ T cells, related to Figure 2B. Frequency of monofunctional (1 cytokine/cell) and polyfunctional (>1 cytokine/cell) CD4$^+$ and Vδ2$^+$ γδ T cells after three immunizations (III) compared to the baseline (B).

See Table S8. Excel file containing additional data too large to fit in a PDF, related to Figure 2B

Table S9: TCM and TEM frequencies among CD4$^+$ and Vδ2$^+$ γδ T cells, related to Figures 2C-E. Frequencies normalized to CD3$^+$ population.

See Table S9. Excel file containing additional data too large to fit in a PDF, related to Figure 2C, 2D and 2E

Table S10: Correlations between targeted antigen counts and T cell population frequency, related to Figures 2F and S4.

See Table S10. Excel file containing additional data too large to fit in a PDF, related to Figure 2F and S4
